# Active Trafficking of Alpha 1 Antitrypsin across the Lung Endothelium

**DOI:** 10.1371/journal.pone.0093979

**Published:** 2014-04-17

**Authors:** Angelia D. Lockett, Mary Beth Brown, Nieves Santos-Falcon, Natalia I. Rush, Houssam Oueini, Amber J. Oberle, Esther Bolanis, Miryam A. Fragoso, Daniela N. Petrusca, Karina A. Serban, Kelly S. Schweitzer, Robert G. Presson Jr., Michael Campos, Irina Petrache

**Affiliations:** 1 Department of Medicine, School of Medicine, Indiana University, Indianapolis, Indiana, United States of America; 2 Department of Physical Therapy, School of Health and Rehabilitation Sciences, Indiana University, Indianapolis, Indiana, United States of America; 3 Department of Anesthesia, School of Medicine, Indiana University, Indianapolis, Indiana, United States of America; 4 Division of Pulmonary and Critical Care Medicine, University of Miami, Miami, Florida, United States of America; 5 “Richard L. Roudebush” Veteran Affairs Medical Center, Indianapolis, Indiana, United States of America; University of Colorado, Denver, United States of America

## Abstract

The homeostatic lung protective effects of alpha-1 antitrypsin (A1AT) may require the transport of circulating proteinase inhibitor across an intact lung endothelial barrier. We hypothesized that uninjured pulmonary endothelial cells transport A1AT to lung epithelial cells. Purified human A1AT was rapidly taken up by confluent primary rat pulmonary endothelial cell monolayers, was secreted extracellularly, both apically and basolaterally, and was taken up by adjacent rat lung epithelial cells co-cultured on polarized transwells. Similarly, polarized primary human lung epithelial cells took up basolaterally-, but not apically-supplied A1AT, followed by apical secretion. Evidence of A1AT transcytosis across lung microcirculation was confirmed in vivo by two-photon intravital microscopy in mice. Time-lapse confocal microscopy indicated that A1AT co-localized with Golgi in the endothelium whilst inhibition of the classical secretory pathway with tunicamycin significantly increased intracellular retention of A1AT. However, inhibition of Golgi secretion promoted non-classical A1AT secretion, associated with microparticle release. Polymerized A1AT or A1AT supplied to endothelial cells exposed to soluble cigarette smoke extract had decreased transcytosis. These results suggest previously unappreciated pathways of A1AT bidirectional uptake and secretion from lung endothelial cells towards the alveolar epithelium and airspaces. A1AT trafficking may determine its functional bioavailablity in the lung, which could be impaired in individuals exposed to smoking or in those with A1AT deficiency.

## Introduction

Alpha-1 antitrypsin (A1AT) is a glycoprotein serine protease inhibitor that is produced and secreted from hepatocytes into the systemic circulation. In hepatocytes, A1AT undergoes N-linked glycosylation and is released through the classical secretory pathway, via processing through the ER and the Golgi apparatus [1], [2]. Lung endothelial cells do not synthesize A1AT, but they actively take up the circulating serpin via endocytosis [3]. Endocytosed A1AT exerts anti-apoptotic effects and modulates inflammatory responses to TNFα in endothelial cells [4]. However, the fate of A1AT internalized by lung endothelial cells is not known.

Endocytosed proteins are processed by the ER/Golgi network, where they can get either glycosylated and secreted extracellularly, or targeted for degradation by the lysosome. Alternatively, certain intracellular proteins can be handled through non-classical secretory pathways, via lysosomes, exosomes formed from multiple vesicular bodies, direct transport from the cytosol to the extracellular space, or by plasma membrane blebbing and vesicle shedding [5]. It is not known which if any of these mechanisms handles A1AT trafficking or transcytosis across the capillary-alveolar membrane. Movement of molecules across the capillary endothelium can occur through bulk-phase transport or the more selective process of receptor-mediated endocytosis and favors apical to basolateral transport because of the concentration gradient on the blood side of the endothelium [6]. We and others have shown that A1AT is taken up primarily by clathrin, but also via caveolae-dependent endocytosis, both of which have been implicated in transcytosis of molecules across the endothelium [3], [7]. Studies analyzing low density lipoprotein (LDL) transport suggest that multiple mechanisms may exist to transport one molecule. Furthermore, the mode of endocytosis may determine the fate of the internalized molecule [6], i.e. sorting for cellular use, degradation, or basolateral secretion. For example, clathrin-dependent LDL uptake leads to transcytosis while caveolae-dependent LDL uptake leads to degradation and release of cholesterol for intracellular use [6], [8], [9]. In the lung, it has been shown that the upper airway epithelium can perform cargo-dependent bidirectional transport [10]–[12], while the lung capillary endothelium can handle bidirectional transcytosis of both albumin and fluid [13]–[15]. No studies of A1AT transcytosis have been described, to the best of our knowledge.

Pulmonary A1AT levels decline in parallel with decreasing circulating levels in A1AT deficiency (AATD), a hereditary disease whereby a point mutation, Glu342Lys [16], [17] causes A1AT polymerization and accumulation in the liver. Individuals affected with AATD are at high risk for COPD, especially if they smoke cigarettes, due to unopposed elastase activation [18], [19] as well as excessive apoptosis [20], [21] and lung inflammation [22]–[24]. A1AT directly protects lungs from elastase, inflammation, and endothelial cell apoptosis, the latter effect requiring active intracellular uptake of A1AT by the endothelium, a step inhibited by CS exposure [3]. In clinical practice, A1AT supplementation via weekly intravenous infusions of purified protein ameliorates lung disease in only a subset of AATD patients with COPD, suggesting further optimization of therapy is needed. Understanding the mechanisms of normal trafficking of A1AT across the endothelium, but also those underlying its disruption, may highlight new risk factors for CS-induced lung disease in both AATD and usual COPD, and may inform future strategies for A1AT supplementation. In this report, we describe that lung microvascular endothelial cells assist in A1AT transcytosis via both classical and non-classical pathways and this process is markedly inhibited by CS exposure.

## Materials and Methods

### Ethics Statement

Research involving normal human bronchial epithelial cells falls under exemption 4 of the Federal Code of regulations (CFR), 45 CFR 46.101(b), since it did not involve human subjects as defined in the CFR.

The experiments utilized mice under the Institutional Animal Care and Use Committee of Indiana University-approved protocol, which is in compliance with the NIH guidelines. The animals were housed in the Indiana University animal facility, which is compliant with the NIH guidelines for veterinary care. Veterinary care was provided to all animals used. Animals were anesthetized with isoflurane, followed by the experimental procedure for intravital microscopy. No discomfort was observed.

### Materials and Reagents

All reagents were from Sigma-Aldrich (St. Louis, MO), unless otherwise specified. Purified A1AT pooled from human plasma was from Sigma-Aldrich or from Baxter Healthcare (Deerfield, IL), as indicated. Tunicamycin was from Enzo Life Sciences (Ann Arbor, MI). Antibodies raised against human A1AT were from Bethyl Laboratories (Montgomery, TX); anti-vinculin was from Calbiochem (La Jolla, CA); and GAPDH was from Cell Signaling (Danvers, MA). Transwell inserts (0.4 µm pore size) with polyethylene terephthalate (PET) membranes and companion plates were from BD Biosciences (San Jose, CA).

### Cell Cultures

Primary rat lung microvascular endothelial cells (RLMVEC), a kind gift from Dr. Troy Stevens (University of South Alabama, Mobile, AL), were maintained in DMEM supplemented with 10% fetal bovine serum and 1% penicillin/streptomycin and treated at 37°C in 5% CO_2_ [25]. Rat lung epithelial cells were from ATCC (Manassas, VA) and maintained in F12 medium containing 10% fetal bovine serum.

### A1AT Secretion from Endothelial Cells

Confluent monolayers seeded on solid tissue culture plates were serum-deprived for 2 h prior to treatment with A1AT (100 µg/mL). Exogenous A1AT was removed from the media by washing (2×5 min) with serum-free media at 37°C. At the end of the experiment, supernatants were collected and floating cells were removed by centrifugation (500× g; 5 min; 4°C). Adherent cells were rinsed once with PBS and harvested in RIPA buffer containing protease and phosphatase inhibitors. Whole cell lysates were collected by incubating cells on ice (30 min) followed by sonication and centrifugation (14,000 rpm; 10 min; 4°C). In a subset of experiments, cells were treated with the N-glycosylation inhibitor tunicamycin either following A1AT washing (84–840 ng/mL; 18 h) or prior to A1AT treatment (420 ng/mL; 1 h), or with the Golgi secretion inhibitor brefeldin A (1 ug/mL; 1 h).

### Endothelial Transcytosis Experiments

Endothelial cells (5×10^5^/well) were seeded on 6-well transwell inserts and grown to confluence (for 48 h). Confluency was confirmed by a) culturing cells on the transwell with culture media added exclusively to the top chamber and checking for its presence (leakage) into the bottom chamber; b) light microscopy; and c) measuring the leakage of FITC-Dextran across the endothelium. Cells were serum-deprived for 2 h prior to treatment with purified human A1AT (100 µg/mL). A1AT transcytosis across the endothelial monolayer (basolateral secretion) was measured by collecting and then immunoblotting for A1AT in the bottom supernatants, as well as whole cell lysates.

### Co-culture Transcytosis Experiments

Rat lung epithelial cells (2.5×10^5^ cells/well) were grown and treated submerged in media, on the bottom of transwell membranes by flipping the insert and allowing cells to attach for 3 h. Endothelial cells were then seeded at 2.5×10^5^ cells/well on the top of the transwell membrane. Following complete confluency of both monolayers (48 h) visualized by light microscopy, endothelial cells were apically treated with A1AT (100 µg/mL) for the indicated time followed by collection of bottom supernatants and whole cell lysates.

### Primary Cultures of Normal Human Bronchial Epithelial Cells (NHBE)

Donor lungs unapproved for transplantation were obtained from the University of Miami Life Alliance Organ Recovery Agency. NHBE cells were isolated via mucosal dissection and protease digestion and expanded in submerged culture. Passage 1 cells were plated onto collagen-coated Transwell clear (T-clear) membrane filter inserts (Costar). After cells reached confluence, the medium from the apical chamber was removed exposing the cells to air. NHBE cells differentiated in these air-liquid interface (ALI) cultures were used for experiments after at least a 3-week exposure to air, when mucus was visually apparent and cilia fully developed. Specific immunostaining confirmed the presence of differentiated ciliated, serous and mucous cells in these cultures.

### Immunofluorescence

Internalization of fluorescently labeled A1AT with Alexa Fluor 488 (Molecular Probes) by NHBE cells was evaluated using a Leica TCS SP5 confocal microscope after cells were fixed with 4% paraformaldehyde. For cell differentiation, cilia were visualized using a mouse anti-human acetylated tubulin IgG (5.6 µg/ml, Sigma) coupled with a Cy5-labeled rabbit anti mouse IgG (15 ng/ml) (Chemicon International, Temecula, CA). Nuclei were visualized with 4′-6-diamidino-2-phenylindole (DAPI).

### A1AT Enzyme Linked Immunosorbent Assay (ELISA)

ELISA quantification of A1AT in cell-free supernatants was performed as follows: protein samples were diluted 1∶1000 in TTBS-1% BSA and placed in 96-well plates (100 µl/well) (Immulon 4HBX, VWR, West Chester, PA). Bovine serum albumin was used as a control and ovalbumin (1%) was used as a blocking agent. Goat anti-human A1AT antibody (25 µg/ml, MP Biomedicals, Irvine, CA) and mouse anti-human secretory leukocyte protease inhibitor (SLPI) IgG (1 µg/ml, Cell Sciences, Canton, MA) diluted in Voller's buffer (15 mM Na_2_CO_3_, 35 mM NaHCO_3_, 3 mM NaN_3_, pH 9.3) at a dilution of 1∶500 was added and incubated overnight at 4°C. The plate was washed and secondary polyclonal peroxidase-conjugated goat anti-rabbit IgG (40 ng/ml, Kirkegaard & Perry Laboratories Gaithersburg, MD), was added for 1 h. Color development was achieved using O-phenylenediamine-dihydrochloride (10 mg in 30 ml of 23.3 mM citric acid, 50 mM Na_2_HPO_4_, pH 5.0). The reaction was stopped with 0.1M H_2_SO_4_ and read at 490 nm in a SpectraMAX PLUS 384 plate reader (Molecular Devices, Sunnyvale, CA). A1AT concentrations were calculated by interpolation with the standard curve. Data are expressed as mean of three independent experiments with standard deviation.

### Treatment of NHBE Cells with Endothelial Cell-secreted A1AT

Endothelial cells grown on transwell inserts were treated with A1AT (2 mg/mL, 2 h), which was then washed out and replaced with media; conditioned media was collected from the apical chamber 2 h later. NHBE cells differentiated at ALI were serum-starved for 2 hours and the apical surface and basal compartment washed and replaced (for 2 h) with endothelial cell-conditioned media. Apical PBS washes and cell lysates were collected and A1AT was quantified by ELISA.

### Preparation of Cigarette Smoke Extract and Treatments

Cigarette smoke extract (CSE) was prepared from research-grade cigarettes (3R4F) purchased from the Kentucky Tobacco Research and Development Center (University of Kentucky, Lexington, KY). 100% CSE was generated by bubbling two cigarettes into 20 mL of PBS (pH 7.4, without calcium and magnesium) at a rate of 1 cigarette/min to 0.5 cm above the filter followed by readjusting the pH to 7.4 and filtration using a 0.22 µm Steri-flip filter (Millipore, Bedford, MA). Air control extract was prepared using a similar procedure by replacing the cigarette with ambient air bubbled into PBS. Endothelial cells were treated with 2.5% CSE diluted in serum-free DMEM.

### Western Blot Analysis

Protein concentration was determined by BCA analysis and equal amounts of protein were resolved by 10% SDS-PAGE, followed by immunoblotting, as previously described [20]. The intensity of bands detected by Western blotting was measured by densitometry using ImageJ software (NIH).

### Endothelial Microparticle Isolation and Characterization

Apical and basolateral supernatants were collected and centrifuged at 1500× g for 10 min. The supernatant was then transferred to ultracentrifuge tubes and microparticles were isolated at 100,000× g for 1.5 h. For analysis by Western blot, the microparticles were resuspended in RIPA lysis buffer containing proteases and phosphatase inhibitors. For flow cytometry analysis, microparticles were resuspended in 0.5 mL of PBS, stained with Nile Red (1 µg/mL; 5 min) at room temperature and counted for 240 s using the Cytomics FC500 cytofluorimeter with CXP software (Beckman Coulter, Fullerton).

### Time-lapse Intravital Imaging of Cultured Cells and Mouse Microcirculation

Cells were stained in serum-free media with the following vital dyes: Bodipy TR C5-ceramide-BSA (5 mM, 30 min; 40°C; Molecular Probes; Invitrogen) in HEPES medium, to visualize the Golgi Complex or Lysotracker Red (50 nM; 30 min; Molecular Probes, Invitrogen), to visualize lysosomes. After washing with warm serum-free media, cells were incubated with Alexa Fluor 488 (Life Technologies, Grand Island, NY)-labeled A1AT (100 µg/ml; 30 min), which was then removed by a second wash with warm serum-free media, and then visualized by live microscopy every 3 minutes for 2 h. Images were acquired using a Perkin-Elmer spinning disk confocal microscope system mounted on a Nikon TE 2000 U inverted microscope, using Nikon ×100 NA 1.4 oil immersion plan apochromatic objective. The excitation wavelength was set at 56 nm or 488 nm, and the emission filters used were 600/45 or 525/50 for all experiments. The system is equipped with an Andor EM-CCD system (South Windsor, CT).

### Intact Animal Preparation

Mice were anesthetized by inhaled isoflurane (5% in oxygen), orotracheally intubated with a 20 gauge catheter and ventilated at a rate of 130 breaths/min. For the remainder of the experiment, general anesthesia was maintained with isoflurane (2% in oxygen). The right internal jugular vein and the carotid artery were cannulated via cut down with a 26 gauge catheter for administration of fluid, A1AT and fluorescent probes, and for monitoring of systemic blood pressure, respectively. A thoracotomy was performed in the 5^th^ left intercostal space and the 6^th^ rib was excised. The window, measuring 0.5 cm in diameter, was interfaced with the lung via this thoracotomy. A Texas Red-Dextran (150 kDa amino dextran;TdB Consultancy, Uppsala, Sweden, conjugated to Texas Red, Invitrogen, Carlsbad, CA) 20–22 mg/kg, was administered intravenously (i.v.) to label the circulating plasma. Nuclei were stained with Hoechst 33258 (Invitrogen; 10–12 mg/kg; i.v.). A1AT was labeled with Alexa Flour 488 (Life Technologies). Intravital two-photon microscopy was conducted on a Zeiss LSM-510 Meta Confocal/Multiphoton Microscope system mounted on an upright Axioplan 2 stand (Carl Zeiss Microimaging, LLC, Thornwood, NY) and a vibration-isolation table. The illumination source was a tunable Titanium-Sapphire laser (using a 10W Millennia diode solid state pump laser) (Spectra Physics Lasers Inc, Mountain View, CA). The band pass filters used for the red and green channels were 605/690 and 525/550, respectively. The excitation wavelength was set at 820 nm for all experiments and a non-discanned mode was utilized. A Zeiss C-Apochromat 40x/1.2 W Korr UV-VIS-IR objective was used for imaging and was warmed with an objective heater (Warner Instruments, Hamden, CT). All image series were collected at a constant pixel dwell time, which yielded a 1.1 s frame time for the frame size of 512×512 pixels (169×169).

### Statistical Analysis

Statistical analysis was performed using Prism (Irvine, CA). The differences between groups were compared using unpaired Student t-test or ANOVA with Student-Newman-Keuls *post hoc* test. Data from independent experiments were expressed as mean ± SEM. Statistically significant difference was accepted at p<0.05.

## Results

### *In Vivo* Trafficking of A1AT

**Figure 1:**
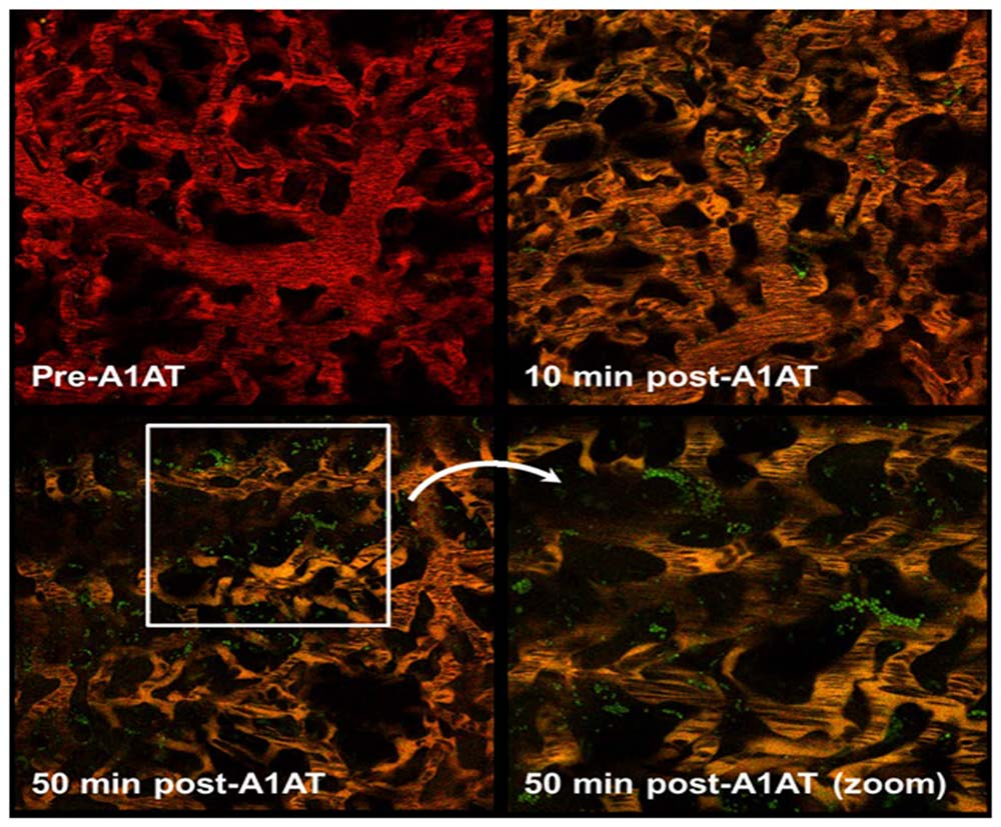
Intravital microscopy of A1AT trafficking across the lung microvascular circulation in the intact mouse. Selected frame from timelapse video of the pulmonary microcirculation following intravenous injections of rhodamine-labeled rat albumin (red) and airspaces (dark) showing no AF488-A1AT (green) prior to injection (pre-A1AT). Note that following AF488-A1AT intravenous administration there is circulating protein (orange microcirculation) as well as A1AT protein in the airspaces (green punctate signal), without evidence of lung edema (no red extravasation), suggesting active transcytosis from the circulation at 10 min and 50 min post injection.

To monitor A1AT trafficking in the pulmonary microcirculation in real time, we employed a method of intravital two photon microscopy in the intact mouse, previously described in detail [26]. Mice were injected via jugular vein with A1AT (10 mg/kg) labeled with Alexa Fluor 488. Immediately following intravenous injection, A1AT co-localized with the TR-dextran-labeled lung microcirculation (Fig. 1). Trafficking of A1AT into the lung interstitium was evident as early as 10 min after A1AT injection, despite lack of plasma extravasation into alveolar interstitium or airspaces (Fig. 1).

### Transcytosis of A1AT in Pulmonary Endothelial and Epithelial Cells

**Figure 2:**
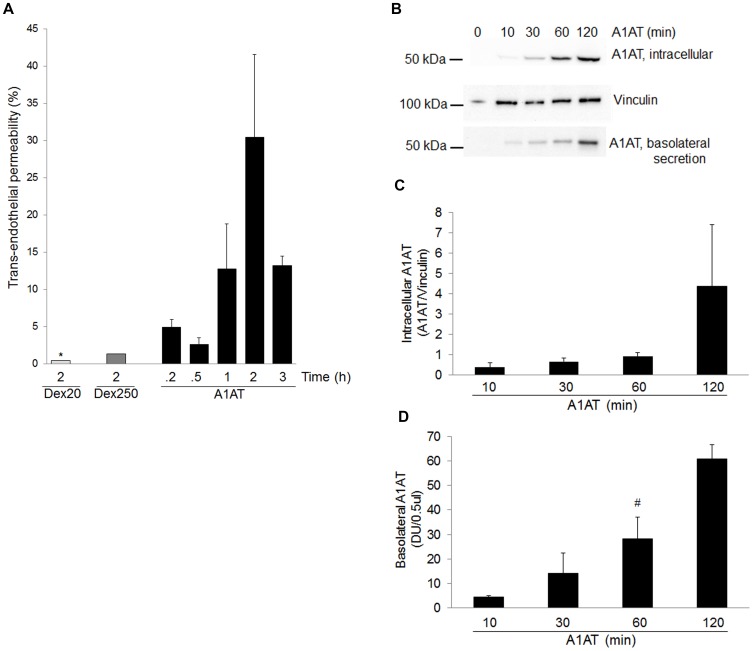
Polarity of A1AT trafficking across cultured pulmonary endothelial monolayers. (A) Fraction (%) of FITC-Dextran (Dex) of 20 kDa (light gray bars) or 250 kDa (dark gray bars) or of A1AT (100 µg/mL; black bars) that crossed confluent endothelial cell monolayers grown on 0.4 µm transwells. (B) Immunoblots of intracellular and basolaterally secreted A1AT from endothelial cells cultured on transwell inserts treated with exogenous A1AT (100 µg/mL; for up to 120 min). (C) Intracellular levels of A1AT quantified by densitometry after normalizing to the vinculin loading control. (D) Transcytosed levels of A1AT normalized to .5 ul of concentrated supernatant. Bars represent mean+SEM; *p = .05 vs A1AT 2 h; #p<.05 vs. A1AT 10 min; n = 3.

**Figure 3:**
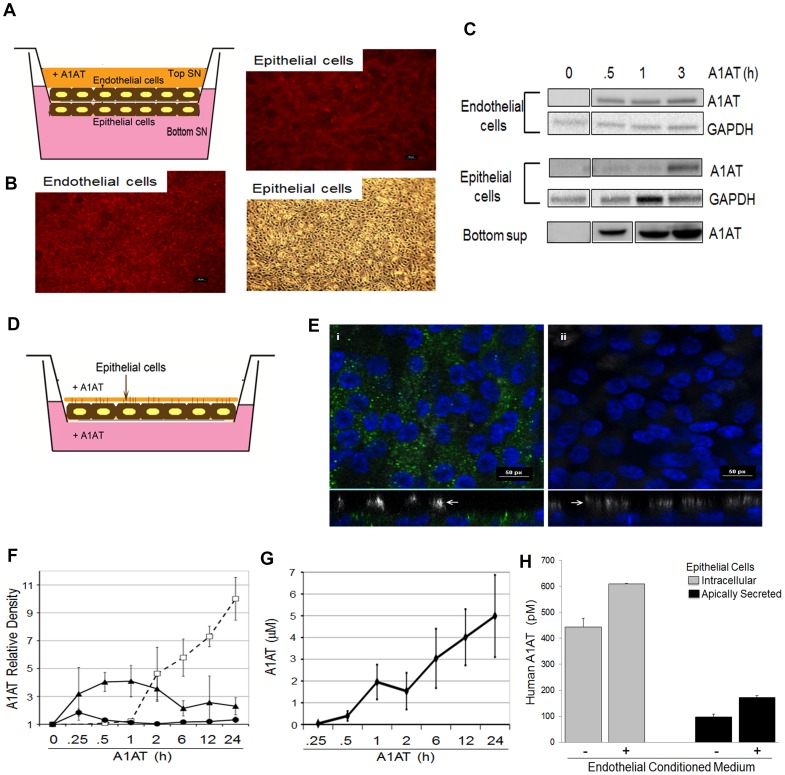
A1AT trafficking across cultured pulmonary endothelial and epithelial bilayers. (A) Co-culture schematic showing pulmonary epithelial cells cultured on the bottom of the transwell membrane and pulmonary endothelial cells cultured on the top. Endothelial cells only were exposed to A1AT. (B) Phalloidin staining (top and epithelial cells) and brightfield microscopy (bottom) showing confluent monolayers of endothelial and epithelial cells seeded on transwell inserts. (C) Immunoblotting of A1AT in cell lysates showing intracellular presence of A1AT in endothelial and epithelial cells and immunoblot of secreted A1AT from concentrated bottom supernatant (representative blots of n = 3). Bands shown are from the same immunoblot. (D). Schematic showing A1AT treatment of the basolateral media or apical surface of NHBE cells differentiated at ALI. (E). Confocal microscopy of NHBE cells differentiated at ALI after 2 h incubation with fluorescently labeled A1AT (green, 20 µM) added to either the basolateral media (i) or apical surface (ii). Only the basolaterally applied A1AT was observed to enter the cells. Arrows indicate ciliated side of the epithelium (cilia stained in white, nuclei in blue). (F). Densitometric quantification of epithelial cell lysates and ASL collected at the indicated times after adding 20 µM of A1AT in either the basolateral (solid line, triangles) or the apical (solid line, circles) compartment from 3 different lung donors measured by Western blotting (mean+SEM; n = 3). Plot (dashed line, squares) showing the relative A1AT present in the ASL after the basolateral application experiment. All fold changes are relative to time 0 before the addition of A1AT. (G) Concentration of A1AT in ASL of ALI cultures after A1AT (20 µM) addition to the basolateral compartment. ASL was collected with 250 µl PBS washes. A1AT quantification was made by customized ELISA and corrected for wash dilution (n = 3 different lung donors). (H) Levels of intracellular and secreted A1AT measured by ELISA (n = 2) in NHBE cells treated with conditioned endothelial media (containing 43.4 nM endothelial-secreted A1AT, 2 h);. n = 1.

Previous work from our laboratory demonstrated that pulmonary endothelial cells, which do not synthesize A1AT, internalize the serpin through an active mechanism of endocytosis [3]. We noted that despite washing out excess A1AT from culture supernatants, we consistently detected human A1AT secreted in fresh serum-free supernatants of rat lung endothelial cells cultured on solid plates (data not shown). We therefore investigated A1AT transcytosis across endothelial cells grown on transwell membranes. Rat lung microvascular endothelial cells were cultured to confluence on transwells and treated with human A1AT. In addition to visual inspection by microscopy, we tested the integrity of the monolayer using FITC labeled- Dextran 20 kDa or -Dextran 250 kDa and compared the permeability to A1AT migration across the membrane. There was only a minimal amount of Dextran recovered in the basolateral supernatants (approximately 1.5%; Fig. 2a). In comparison, there was up to 30% of A1AT that transmigrated from the top to the bottom chamber in the same time span of 2 h, suggesting that the endothelial monolayer exerted proper barrier function and that A1AT did not cross the membrane by simple diffusion. There was a time-dependent increase in the intracellular A1AT in endothelial cells and in the basolateral compartment, indicating protein transcytosis (Fig. 2b–d). Regardless of whether A1AT was added to the apical or the basolateral side of the endothelium, the protein could be detected intracellular and secreted across the trans-well membrane (Fig. S1), suggesting active bidirectional transport of A1AT across the lung endothelium. We next increased the complexity and physiological relevance of the barrier by adding to the endothelial cell monolayer an epithelial cell monolayer. The monolayers were co-cultured until reaching full confluence, on opposite sides of the transwell membranes (Fig. 3a–b). After treatment with human A1AT on the apical aspect of endothelial cells, we detected intracellular A1AT in both endothelial and in the epithelial cells on the opposite side of the transwell, and noted evidence of transcytosed A1AT across the epithelium, in the media from the bottom compartment (Fig. 3c; Fig. S2).

Since A1AT uptake by epithelial cells grown in submerged cultures could have occurred on their apical side via transcellularly “leaked” A1AT, we sought to specifically determine if A1AT uptake by lung epithelial cells is polarized. For this, we exposed NHBE, which were well-differentiated at the air-liquid interface (ALI), to fluorescently tagged A1AT (Fig. 3d). We observed that bronchial epithelial cells only internalize A1AT from the basolateral surface and not from the apical (airway luminal) side (Fig. 3e). We measured the kinetics of A1AT uptake by Western blotting and noted that intracellular A1AT was apparent 15 minutes after treatment with peak uptake at 1 h, although it could be detected for up to 24 h after treatment (Fig. 3f). We next determined if A1AT can transcytose through the lung epithelial layer to the airway surface liquid (ASL). The supply of physiological concentrations of A1AT (20 µM) typically circulating in healthy, non-AATD individuals [27], caused a relatively slow rise in A1AT concentration in ASL, reaching approximately 5 µM at 24 h (corrected for dilution, Fig. 3g). The slow initial rise in ASL A1AT concentrations reflects epithelial internalization of A1AT with subsequent release, which occurred concurrent with progressive decrease in A1AT internalization (Fig. 3f). We next studied if A1AT secreted from pulmonary endothelial cells can be taken up and released from lung epithelial cells. We collected the apical endothelial conditioned media prepared as described in the Methods section following 2 h exposure to exogenous A1AT. Human lung epithelial cells grown at ALI were then exposed baso-laterally to either control serum-free or to conditioned medium from A1AT-treated endothelial cells and then intracellular and secreted A1AT were measured by ELISA. Compared to untreated epithelial cells that synthesize their own A1AT intracellular and secrete it extracellular at baseline, epithelial cells exposed to endothelial-conditioned media had increased concentrations of both intracellular and secreted A1AT (Fig. 3h). While it remains to be determined whether the excess A1AT is due to exogenous protein trafficking or increased endogenous production, these observations suggest unidirectional transport of A1AT from the circulation to the ASL with active participation of the pulmonary endothelium and epithelium.

### Cigarette Smoke Exposure Inhibits A1AT Transcytosis

**Figure 4:**
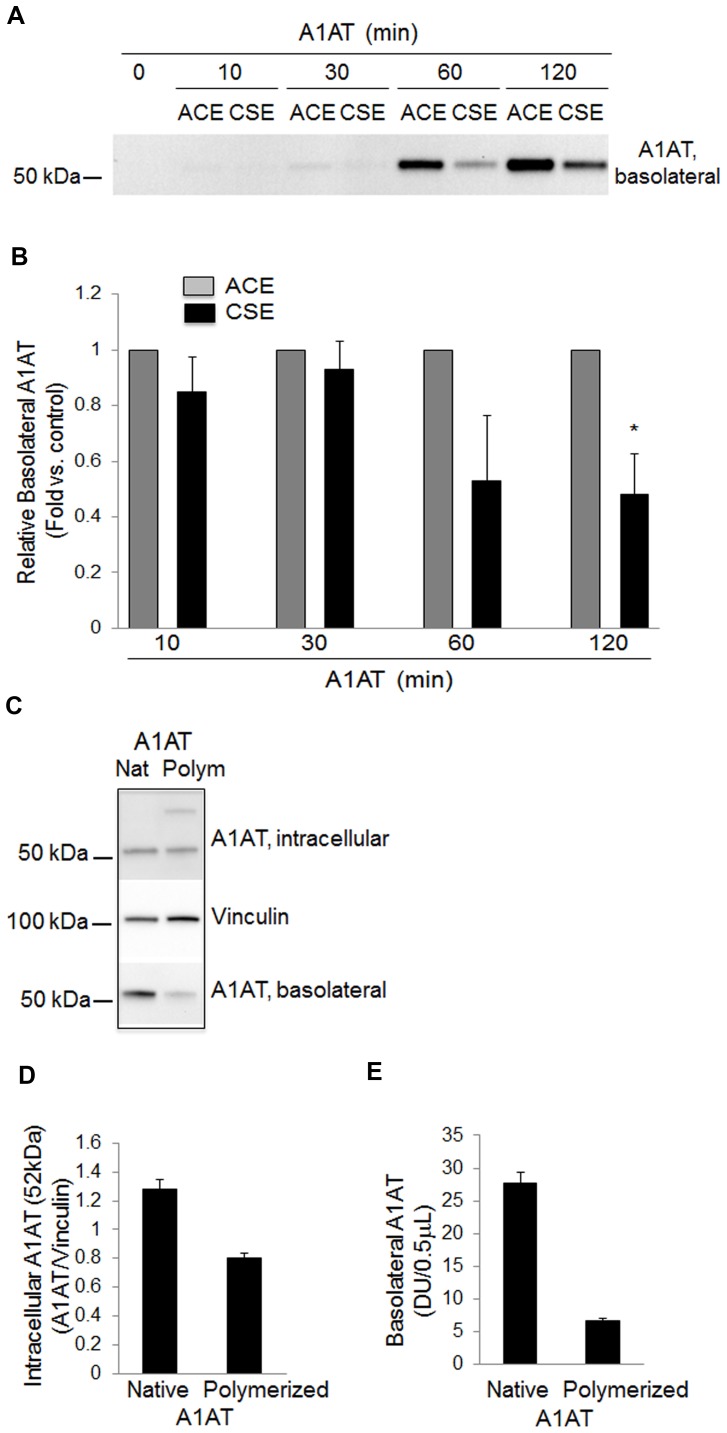
Effect of cigarette smoke extract exposure on A1AT transcytosis across cultured lung endothelial monolayers. (A) Immunoblot of A1AT from concentrated supernatants showing basolaterally secreted A1AT in endothelial cells co-treated with A1AT (100 µg/mL) and CS or AC extract (2.5%) for up to 120 min (representative blot of n = 3). (B,E) Transcytosed A1AT levels in concentrated supernatants. Bars represent mean+SEM; *p<.05 vs. respective AC; n = 3. (C) Immunoblots of intracellular and basolaterally secreted A1AT in endothelial cells treated with native (Nat) or polymerized (Polym, heated at 60°, 2 h) A1AT (100 µg/mL; Baxter Healthcare). (D) Intracellular levels of A1AT (52 kDa) quantified by densitometry and normalized to the vinculin loading control. n = 2.

In addition to inducing oxidation and polymerization of A1AT [28], CS exposure inhibits A1AT endocytosis by endothelial cells [3]. We determined the effect of exposure to soluble components of CS (that may be absorbed into the circulation) on A1AT transcytosis by exposing cells simultaneously to CS extract (CSE), or a control extract of ambient air, and to human A1AT, for up to 2 h. Compared to control conditions, CSE exposure decreased the levels of transcytosed A1AT (Fig. 4a, b). Similarly, polymerized A1AT showed markedly reduced transcytosis by lung endothelial compared to the native protein (Fig. 4c–e).

### Secretory Pathways for A1AT in Lung Endothelial Cells

**Figure 5:**
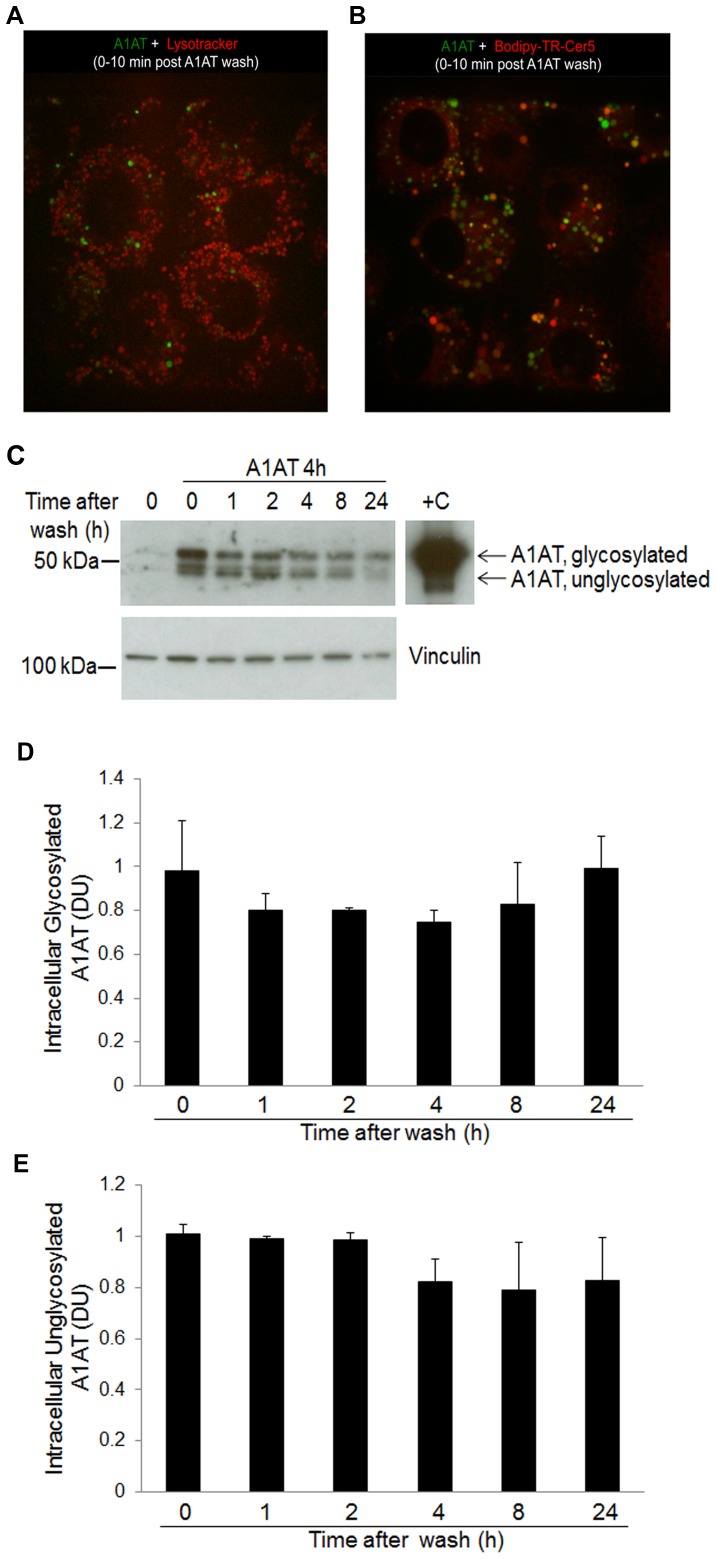
Intracellular trafficking of A1AT to the Golgi system in lung endothelial cells. (A–B) Still frames from time-lapse (10 min) two-photon imaging of A1AT-treated (2 h) lung endothelial cells showing lack of co-localization of labeled A1AT (green) with lysosomal marker, Lysotracker (red; in A), and multiple areas of colocalization (in orange) with a Golgi marker (red, in B). (C) Immunoblot of A1AT in cell lysates of endothelial cells treated on regular tissue culture plates with A1AT (100 µg/mL; 4 h), followed by washing residual extracellular A1AT and harvesting up to 24 h later. Vinculin was used as a loading control for the whole cell lysates. Note time-dependent increase of intracellular glycosylated A1AT. Bands are from the same immunoblot. (D–E) Densitometry of glycosylated and unglycosylated bands of A1AT from immunoblots normalized to vinculin (mean+SD; n = 2).

**Figure 6:**
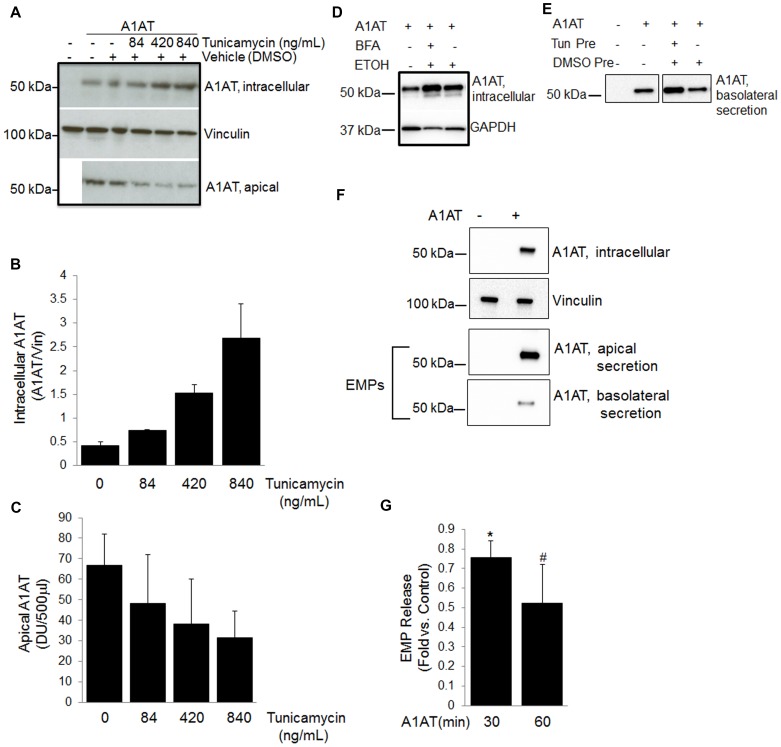
Secretory pathways leading to A1AT transcytosis across cultured pulmonary endothelial monolayers. (A–D) Immunoblot showing effect of inhibition of classical secretory pathway with tunicamycin (A–C; at the indicated doses; 18 h) or brefeldin A (D; 1 µg/mL, 60 min) on intracellular A1AT (A, B, D) and secretion of A1AT (A, C) detected by Western blotting and quantified by densitometry, using vinculin as loading control. Secreted A1AT was measured in equal volumes of supernatants (which were concentrated 25-fold). (E) Pre-inhibition of the classical secretory pathway with tunicamycin (1 h) enhances A1AT (3 h) basolateral secretion, measured by Western blotting of concentrated supernatants, suggesting the utilization of a non-classical secretory pathway. Bands are from the same immunoblot. (F) Alternative secretion of A1AT (100 µg/mL, 2 h) by endothelial cells via microparticles release, as detected by Western blotting of A1AT in endothelial microparticles isolated from supernatants via ultracentrifugation (representative blot of n = 3). (G) Time course of basolateral EMP release from endothelial cells treated with A1AT (100 µg/mL). Bars represent mean+SEM; *p<.05, #p = .07 vs. control; n = 3.

We used time-lapse microscopy to determine the fate of A1AT, once endocytosed by endothelial cells, focusing on its colocalization with either the lysosome, which could indicate degradation, or the Golgi apparatus, which would suggest further processing. Using the vital dye Lysotracker and fluorescently-labeled A1AT, we did not detect co-localization of A1AT with lysosomes during 10 minutes of tracking of A1AT movement that followed washing out exogenous A1AT (Fig. 5a; Movie S2). In contrast, A1AT co-localized with the vital dye Bodipy TR C5-ceramide-BSA, suggesting processing by the Golgi apparatus (Fig. 5b; Movie S1), such as glycosylation and secretion. To determine if A1AT glycosylation occurs in the endothelium, we used SDS-PAGE to track the movement of slow migrating (glycosylated) and fast migrating (unglycosylated) A1AT bands after A1AT treatment for 4 h, followed by washing out the treatment medium. Immediately after washing (time 0), both glycosylated and unglycosylated A1AT were noted intracellular. During a 24 h time-course, the levels of intracellular unglycosylated A1AT mildly decreased while the levels of intracellular glycosylated A1AT mildly increased, indicating that A1AT might undergo glycosylation in the lung endothelium (Fig. 5c–e; Fig. S3). Furthermore, inhibition of N-linked glycosylation with tunicamycin dose-dependently increased the intracellular accumulation of A1AT and decreased its secretion in cell culture supernatants (Fig. 6a–c). Similarly, brefeldin A, an inhibitor of secretory vesicles formation in the Golgi, increased the intracellular retention of A1AT (Fig. 6d). These results support an active intracellular glycosylation of A1AT in the Golgi, followed by secretion via the classical pathway.

### A1AT Transport via Endothelial Microparticles

Unexpectedly however, inhibition of N-linked glycosylation with tunicamycin *prior* to A1AT administration increased A1AT transcytosis (Fig. 6e; Fig. S4), suggesting that non-classical transport mechanisms may also be involved in A1AT transcytosis, at least when classical transport is unavailable or inhibited. Such a non-classical secretion of A1AT may occur via microvesicles or microparticles. We isolated endothelial microparticles (EMPs) from the top and bottom supernatants of cells seeded on transwell membranes and treated with A1AT. Both apically secreted A1AT and transcytosed A1AT could be detected in EMPs indicating that A1AT secretion may also occur via this mechanism (Fig. 6f). Interestingly, A1AT inhibited the number of EMP released by lung endothelium (Fig. 6g), but the significance, mechanism, and cell-specificity of this intriguing effect of A1AT remain to be elucidated.

## Discussion

Our results indicate that endothelial cells not only take up A1AT, but they actively process it and release it to the microenvironment, via directional secretion apically and basolaterally. This transcellular traffic occurs in unstimulated basal states and may provide epithelial cells and the alveolar interstitium with protective concentrations of A1AT. We have previously shown that pulmonary endothelial cells internalize A1AT via endocytosis [3] and that once internalized, A1AT protects the cell from apoptosis and prevents endothelial cell propagation of pro-inflammatory signaling by inhibiting TNFα secretion via inactivating TNFα converting enzyme (TACE) [4]. It would be of interest to determine if there are separate pools of A1AT, e.g. that exert protective intracellular functions vs. targeted for glycosylation and secretion. Although we could not detect A1AT co-localizing with lysosomes during unstimulated conditions, we have previously shown that A1AT intracellular levels are decreased by TNFα, via calpain activation, suggesting that, at least during inflammation, A1AT can be degraded intracellular [4].

The recovery of A1AT inside epithelial cells grown adjacent to the basolateral aspect of endothelium following apical A1AT treatment of endothelial cells, suggests the possibility of transcellular transfer or trafficking of A1AT across the capillary-alveolar barrier. This finding may also explain why A1AT can be recovered in the bronchoalveolar lavage fluid in quantities higher than would be predicted by secretions from epithelial cells or airway inflammatory cells [29], [30]. Such a functional connection between endothelial cells and epithelial cells may be involved in signal exchange. The importance of such a cellular crosstalk has emerged in several reports on intercellular membrane transfer [31]–[34]. Cells can communicate by direct cell-cell contact [35] or via mechanisms involving cellular membrane-derived structures such as membrane vesicles or microparticles [36], [37], exosomes [38], [39], apoptotic bodies [40], tunneling nanotubes [41], [42], and cytoneme or filopodial bridges [43]. By intercellular communication, one cell type can transfer proteins to cells of a different type, proteins which are not normally transcribed by the recipient cells, thus endowing recipient cells with new properties [44]. Endothelial microparticles, in particular, can transfer proteins and microRNA. We detected transcytosed A1AT in EMPs, despite an inhibitory effect of A1AT on EMP release by endothelial cells. Since, A1AT inhibits endothelial cell apoptosis, a focus of future studies will be to characterize these EMPs and determine if A1AT preferentially inhibits production of apoptotic EMPs.

Our results indicated bidirectional transcytosis of A1AT across the endothelium and unidirectional transcytosis across the epithelium (basolateral to apical), suggesting A1AT transcytosis mechanisms may be cell type-specific. This is further supported by a previous report that native A1AT is transcytosed apically in kidney (MDCK) epithelial cells [45]. Furthermore, a fusion protein consisting of a polymeric immunoglobulin receptor linked to human A1AT was effectively transported in a basolateral-to-apical direction across in vitro model systems of polarized respiratory epithelium [46].

The traffic across endothelium of abnormal structural and functional A1AT forms (e.g. oxidized, reactive center loop-cleaved, polymerized) present in ZZ individuals or generated by cigarette smoking and free reactive oxygen species in MM individuals has not been reported, to our knowledge. These abnormal A1AT forms exhibit impaired canonical (e.g. anti-neutrophil elastase activity) as well as non-canonical (e.g. anti-inflammatory and anti-apoptotic) functions. We noted that CSE inhibits transcytosis of A1AT, by a yet unidentified mechanism. CS causes ex-vivo oxidation and augments polymerization of plasma and bronchoalveolar fluid A1AT from ZZ individuals (Z-AAT) and, at a lower rate, from MM individuals (M-AAT) [28]. Oxidized A1AT has decreased uptake by lung endothelial cells and CSE exposure weakens the clathrin mediated-uptake machinery [3]. However, we also noted decreased secretion of already taken up A1AT in endothelial cells exposed to CSE or those directly exposed to polymerized A1AT. Potential mechanisms by which secretion of A1AT may be diminished by CSE could include abnormal misfolding of the oxidized A1AT or aberrations in calnexin phosphorylation levels, since in hepatocytes, calnexin phosphorylation promotes endoplasmic reticulum quality control which slows the secretion rate of misfolded A1AT [47].

These studies indicate that endothelial cells may act as an active conduit for A1AT secretion and transcytosis across its barrier. Transcytosed A1AT may be secreted directly to the lung interstitium and/or taken up by lung epithelial cells. Cigarette smoking inhibits A1AT transcytosis and may decrease A1AT availability in the lung interstitium or epithelium, potentially reducing its protective effects. Since A1AT can be secreted through both classical and non-classical secretory pathways, further investigations are necessary to determine the role of each specific secretory pathway on the function of A1AT. The effect of CS on epithelial uptake, processing and secretion of A1AT also deserves further study. Our observation that endothelial cells exhibit bidirectional secretion of A1AT when exogenous A1AT is removed from the cells, suggests that the concentration gradient of A1AT on the apical side of the cell may determine the directionality of A1AT release. This could be of particular importance in AATD patients who have low circulating concentrations of A1AT which could, in turn, contribute to lower lung A1AT levels. Elucidating the mechanism by which A1AT is taken up and secreted by lung endothelial cells may provide useful targets of therapeutic intervention that can improve A1AT uptake and deliver effective lung concentrations in AATD patients.

## Supporting Information

Figure S1 **Bidirectional transport of A1AT across the pulmonary endothelium.** A. Immunoblots of intracellular and secreted A1AT in endothelial cells treated on the apical (api) or basolateral (baso) surface with A1AT (100 µg/mL, 2 h). B. Percent of A1AT, applied apically or basolaterally that crosses confluent endothelial cell monolayers grown on 0.4 µM transwell membranes.
(TIF)

Figure S2 **Uncut immunoblots of** **Fig. 3** **.**
(TIF)

Figure S3 **Uncut immunoblots of** **Fig. 5** **.**
(TIF)

Figure S4 **Uncut immunoblots of** **Fig. 6** **.**
(TIF)

Movie S1 (AVI)

Movie S2 (AVI)
