# Supplementary figures and images for: Active Trafficking of Alpha 1 Antitrypsin across the Lung Endothelium
Source: PLoS One. 2014 Apr 17;9(4):e93979. doi: 10.1371/journal.pone.0093979 (PMC3990572; doi:10.1371/journal.pone.0093979)

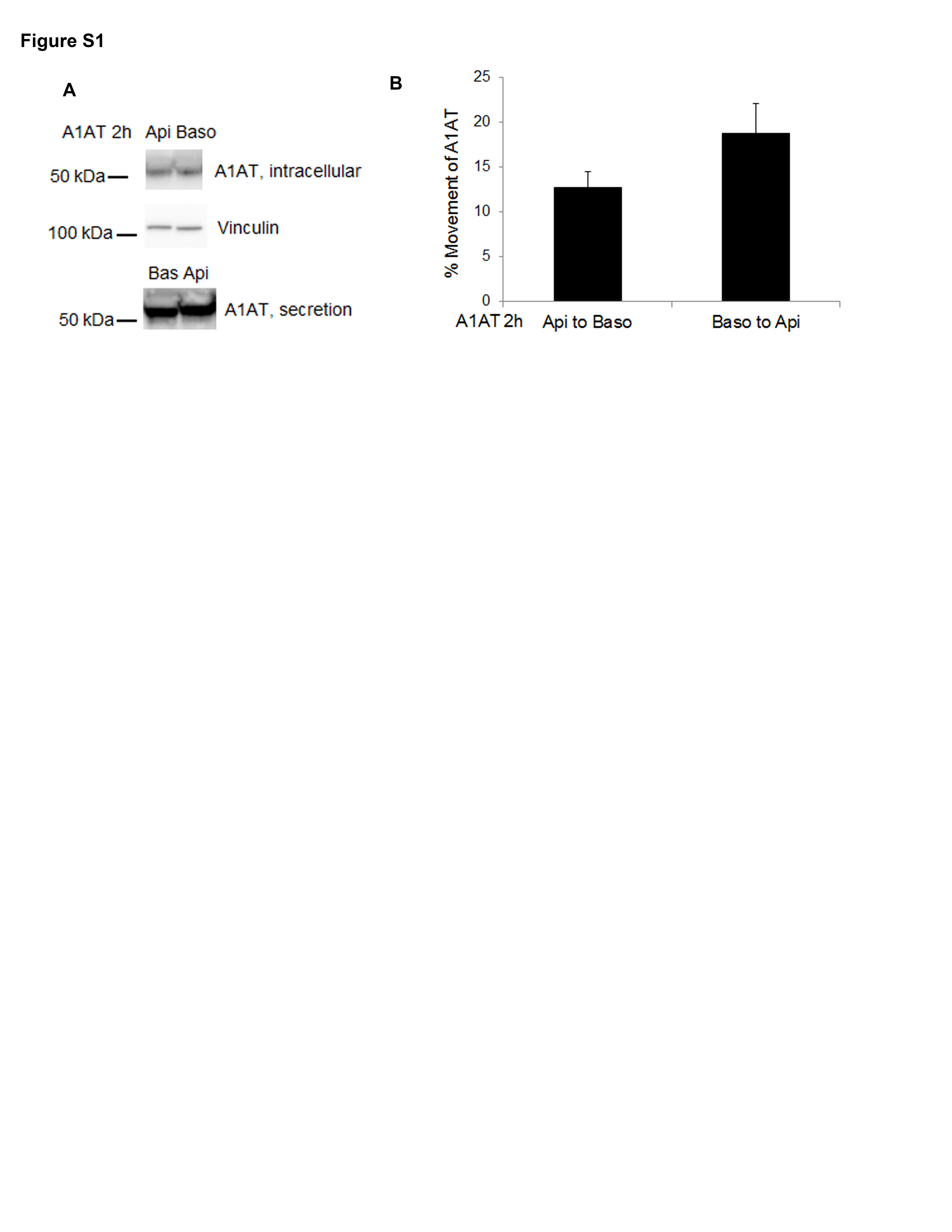

Supplement: Figure S1 — Bidirectional transport of A1AT across the pulmonary endothelium. A. Immunoblots of intracellular and secreted A1AT in endothelial cells treated on the apical (api) or basolateral (baso) surface with A1AT (100 µg/mL, 2 h). B. Percent of A1AT, applied apically or basolaterally that crosses confluent endothelial cell monolayers grown on 0.4 µM transwell membranes. (TIF) [file pone.0093979.s001.tif]

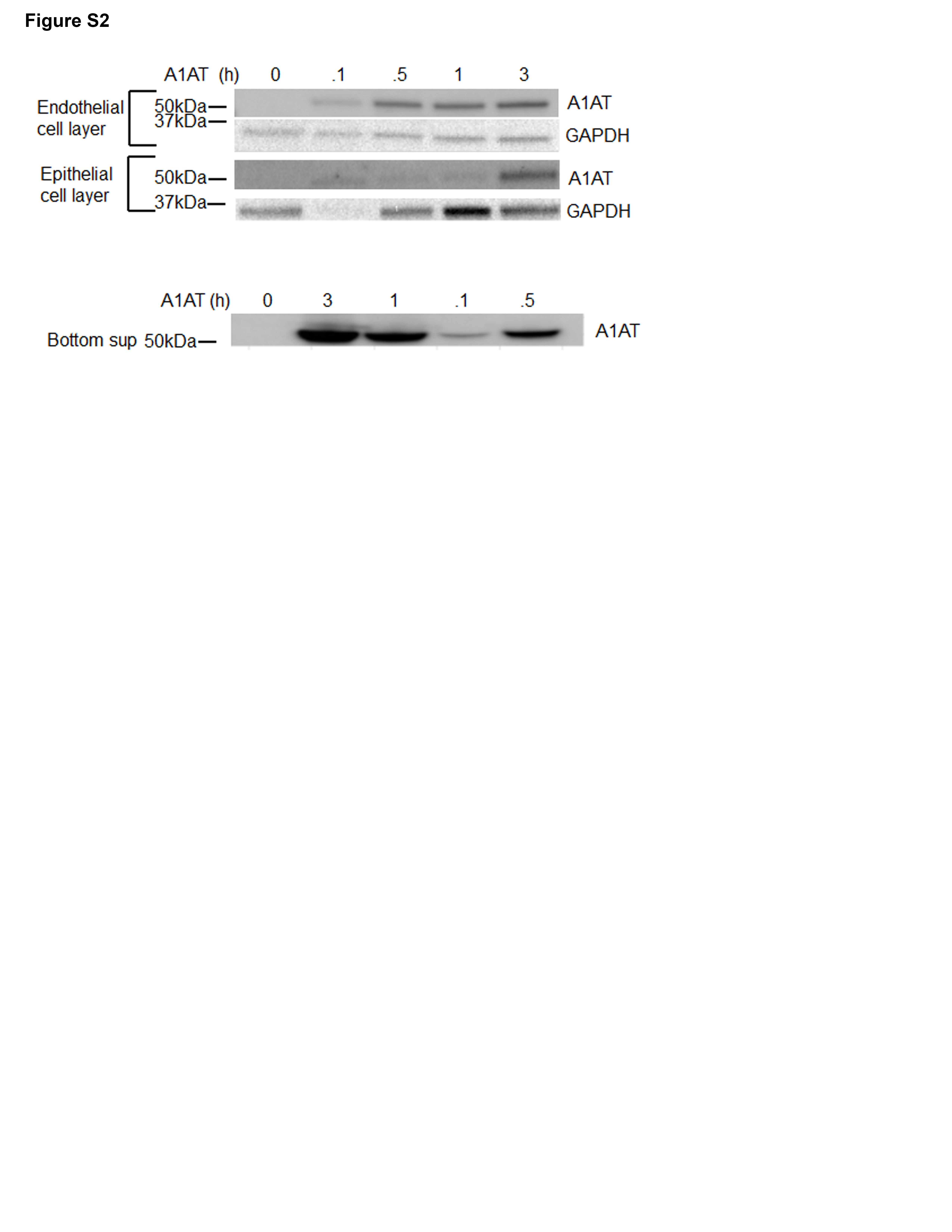

Supplement: Figure S2 — Uncut immunoblots of Fig. 3 . (TIF) [file pone.0093979.s002.tif]

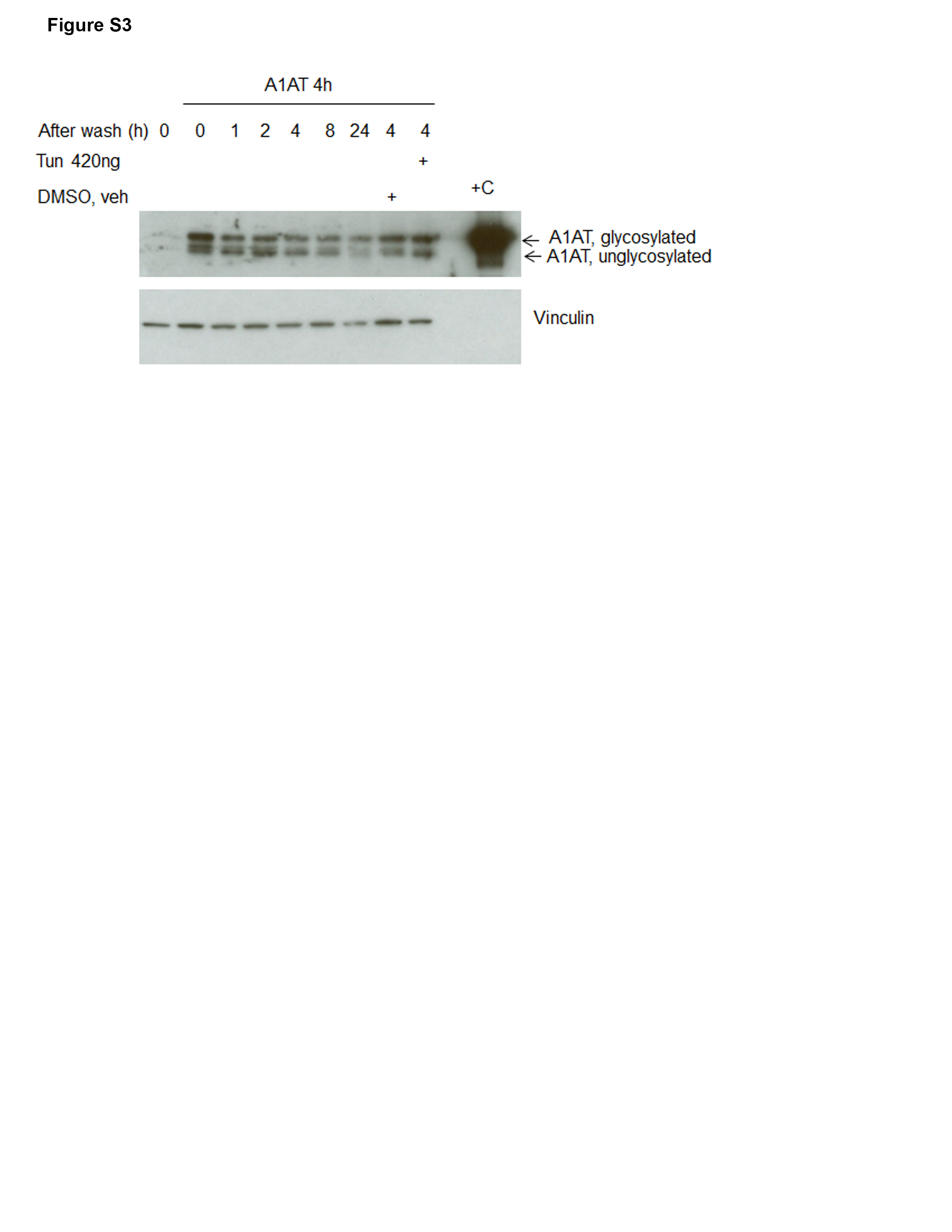

Supplement: Figure S3 — Uncut immunoblots of Fig. 5 . (TIF) [file pone.0093979.s003.tif]

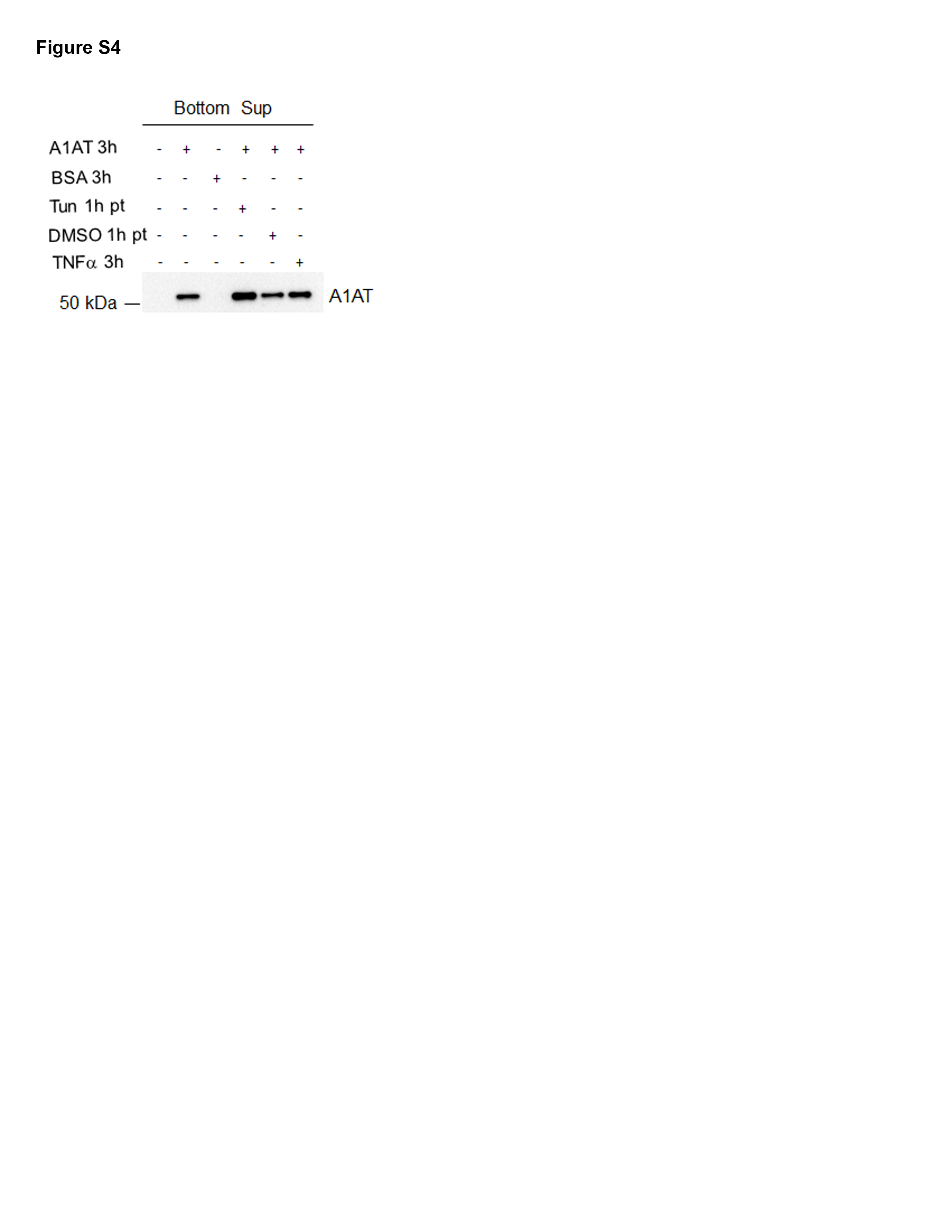

Supplement: Figure S4 — Uncut immunoblots of Fig. 6 . (TIF) [file pone.0093979.s004.tif]
